# Varicella-Zoster Meningitis in an Immunocompetent Male without Fever or Rash

**DOI:** 10.1155/2021/9940393

**Published:** 2021-05-06

**Authors:** Raju Raghunathan, Qasim Khalil, Mohamad Mooty

**Affiliations:** ^1^Department of Hospital Medicine, Cleveland Clinic Abu Dhabi, Abu Dhabi, UAE; ^2^Department of Infectious Diseases, Cleveland Clinic Abu Dhabi, Abu Dhabi, UAE

## Abstract

Varicella-Zoster virus (VZV) is a human herpesvirus that primarily causes chickenpox and can reactivate later in life. Chickenpox occurs mostly in children and is characterized by a typical generalized vesicular rash. Following the primary infection, VZV can remain latent and can reactivate decades later to produce Zoster, being more common in the elderly as well as immunosuppressed individuals. The diagnosis of both the primary and reactivation is mostly clinical from the typical rash. However, when presentations are atypical, it leads to diagnostic challenges. We report an unusual case of VZ reactivation in an immunocompetent young adult presenting without fever, zoster rash, or neuralgia. The diagnosis was established by a positive polymerase chain reaction (PCR) performed on cerebrospinal fluid samples. The patient was treated with acyclovir and responded very well. The diagnosis of VZ meningitis is challenging in the absence of typical features of Zoster rash and requires a high index of suspicion.

## 1. Background

Varicella-Zoster virus (VZV) is a human herpesvirus that primarily causes chickenpox (varicella) and can reactivate later in life [1]. Chickenpox occurs mostly in children and is characterized by a typical generalized, pruritic, vesicular rash. Following the primary infection, VZV can remain latent in neural tissues and can reactivate decades later to produce Zoster (Shingles), which is characterized by a typical localized cutaneous eruption in dermatomal distribution accompanied by neuralgic pain. Zoster (reactivation) is more common in the elderly as well as immunosuppressed populations [2]. Zoster, other than painful rash, can lead to other significant neurological complications [3], severity of which is more in immunosuppressed individuals. Hence, early diagnosis is prudent to prevent morbidity. The diagnosis of both the primary and reactivation is mostly on a clinical basis from the typical rash [3, 4]. However, when presentations are atypical, it may lead to diagnostic challenges. We report an unusual case of VZ reactivation in an immunocompetent young adult presenting with meningitis without evidence of fever, zoster rash, or neuralgia.

## 2. Case Presentation

A 32-year-old male presented to our emergency department with a 2-day history of severe headache. The headache originated in the temporoparietal region and extended to his eyes, causing an inability to focus. He reported it as the worst headache of his life which improved with analgesics. Reported associated symptoms included photophobia, phonophobia, and dizziness. History was negative for any prodromal symptoms of fever, skin rash, nausea, vomiting, diarrhea, neck stiffness, or other neurological deficits. He did not have any dermatomal pattern of pain or rash, at presentation or later in the course of the illness. His past medical history is significant for chickenpox as a child and Crohn's disease. With Crohn's disease, he used to be on immunomodulators and steroids, which he self-discontinued 4 years ago. He reports being symptom-free and in remission. Of note, he was not on any medications that could potentially cause immunosuppression for the last 4 years.

Clinical examination showed normal vital signs, dermatological examination did not reveal any generalized or dermatomal rash, and no vesicles in oral cavity, tongue, or external auditory canal. No conjunctival erythema or discharge.

Given the severe headache, CT head was done to rule out intracranial bleed, which was unremarkable. His inflammatory markers were not significantly elevated, with a white cell count of 9.22 × 10^9^ (4.5–11 × 10^9^), C-reactive protein (CRP) of 6.1 mg/L (<5 mg/L), and an erythrocyte sedimentation rate (ESR) of 18. Lumbar puncture (LP) done, on the day of presentation, showed normal cerebrospinal fluid (CSF) opening pressure, clear in appearance, 139 nucleated cells with 100% lymphocytes, elevated protein 0.53 g/L (0.15–0.45 g/L), and normal CSF glucose of 3.44 mmol/L (2.22–3.89 mmol/L). The CSF study was consistent with aseptic meningitis. CSF culture was negative for any bacterial growth. Meningitis and encephalitis PCR panel (Biofire film array multiplex PCR assay) was positive for VZV. Thus, a diagnosis of Varicella-Zoster meningitis was made.

He received intravenous acyclovir for 4 days [5], symptomatically improved, and hence was discharged with oral valacyclovir for a total of 14 days [5] with close outpatient follow-up.

## 3. Follow-Up

He was reviewed in the clinic after 14 days and a follow-up telephonic encounter was done after 1 year. He was doing well, without any sequelae.

## 4. Discussion

VZV is an exclusively human, alpha herpesvirus which shows neurotropism [1, 6]. Primary infection by VZV causes chickenpox. During primary infection, VZV disseminates hematogenously and infects ganglionic neurons. After the primary infection, the virus can remain latent in neural ganglia throughout the neuroaxis including dorsal root ganglia, cranial nerve ganglia, and also autonomic ganglia of the enteric nervous system [6].

Reactivation can occur decades later and is observed with advancing age and immunosuppressed conditions. Reactivation manifests as zoster or shingles and it may or may not be associated with other neurological manifestations, such as cranial nerve palsies, meningoencephalitis, cerebellitis, myelopathy, multiple ocular disorders, and vasculopathy that can resemble giant cell arteritis [1–3]. VZ meningitis can occur in both immunosuppressed as well as immunocompetent patients with rash [7–9]. There have been multiple case reports of late-onset rash [10]. All neurological complications can occur in the absence of rash [1, 4, 11]. There are reports of varicella meningitis in children who received the live varicella vaccine [12]. However, reports of VZ meningitis without rash and fever in immunocompetent adult patients are uncommon in the literature. Unique aspects of our case are the fact that our patient is an immunocompetent, young adult who, presented without a rash and fever. The only symptom that patient had was the worst headache of life with evidence pointing towards meningeal irritation in the form of photophobia and phonophobia. Clinically, the patient also did not have neck stiffness, as is seen with aseptic/viral meningitis.

In the absence of rash, the clinical diagnosis of VZ meningitis can become a challenge, especially in an immunocompetent patient. Rapid and accurate diagnosis of central nervous system infections is crucial to determine the appropriate treatment. Delay in starting the appropriate therapy has the potential to increase morbidity. Treatment of Varicella meningitis is with oral or intravenous acyclovir [5]. Seriously ill patients should receive intravenous acyclovir (15–30 mg/kg per day in three divided doses), which can be followed by an oral drug once clinically improved. Oral drug therapy can be acyclovir (800 mg five times daily), famciclovir (500 mg TID), or valacyclovir (1000 mg TID) for a total course of 7–14 days [5].

VZ is common in patients with inflammatory bowel disease, who are on medications that reduce immunity [13]. Our patient is an immunocompetent male who presented with headache and photophobia, without rash or fever. The rapid diagnosis was achieved with a timely CSF study and CSF PCR. He was started on appropriate therapy with the resolution of symptoms without any residual findings. He did not develop any rash during the illness and treatment period. Because of its low incidence, atypical presentation, potential significant complications, VZ meningitis without rash requires a high index of suspicion for a timely diagnosis and treatment.

## Additional Points

*Highlights*. (1) All neurological complications of Herpes zoster can occur without a rash. (2) Rapid diagnosis of Zoster-related neurological complications is achieved through CSF PCR assay. (3) Varicella meningitis with mild symptoms can be treated with oral alternatives after initial intravenous acyclovir, for a total duration of 10–14 days [5]. (4) Delay in starting appropriate therapy has the potential to increase morbidity.
